# P08 The quality of life and cost benefits of domiciliary 24-hour piperacillin/tazobactam 13.5 g infusion in patients with necrotizing otitis externa

**DOI:** 10.1093/jacamr/dlac004.007

**Published:** 2022-02-16

**Authors:** Y. Sacranie, N. Fleming, W. Smith

**Affiliations:** Kettering General Hospital NHS Trust, Kettering, Northamptonshire, NN16 8UZ, UK; Kettering General Hospital NHS Trust, Kettering, Northamptonshire, NN16 8UZ, UK; Kettering General Hospital NHS Trust, Kettering, Northamptonshire, NN16 8UZ, UK

## Abstract

**Background:**

The incidence of necrotizing otitis externa (NOE) is increasing with the rise in the population who are elderly or immunocompromised. Currently there is no consensus on an antibiotic regimen for NOE but in a recent survey of otolaryngologists, 90% recommended using IV antibiotics.^1,2^ An initial 6 week period of IV antibiotics such as piperacillin/tazobactam has been recommended, this necessitated a prolonged hospital stay in patients who could be managed at home. To enable domiciliary treatment, a patient pathway was set-up, a ‘mid-line’ inserted and once clinically well (after 0–10 days in hospital) home antibiotic treatment was commenced.

**Objectives:**

To evaluate the patient satisfaction, safety and cost benefit of using 6 weeks of domiciliary piperacillin/tazobactam 13.5 g administered with infusion pump in patients with NOE.

**Methods:**

Since September 2017 to present, 11 patients (9 males, 2 females aged 52–91 years) received domiciliary IV antibiotics for NOE, patients completed a patient satisfaction questionnaire [Glasgow Benefit Inventory (GBI), a validated questionnaire that is used to assess the impact of any clinical intervention]. The cost of this service was compared with the traditional 6 week stay in hospital for IV antibiotic therapy. Patients were only asked to complete the GBI to evaluate a recognized treatment.

**Results:**

The GBI scale ranges from −100 (maximal negative benefit) to 0 (no benefit), to +100 (maximal benefit). Table 1 demonstrates that domiciliary IV antibiotics have a positive impact on total and general health but the low scores for social and physical health reflect worsening of quality of life in patients with NOE. All patients expressed they preferred being at home rather than being in hospital. 6 weeks inpatient care (at £250 per night) equates to £10 500 per patient. Piperacillin/tazobactam 4.5 g injection, costing £7.85 per ampoule, given thrice daily for 6 weeks costs £989. Domiciliary treatment using piperacillin/tazobactam 13.5 g infusion device is £98.16 daily, 6 weeks antibiotic costs £4122 per patient. A domiciliary nurse visit is £120 equates to nursing costs of £5040 for antibiotic given by infusion. Domiciliary potentially saves £2327 per patient.

**Conclusions:**

Although studies have assessed community IV antibiotic service;^3^ this study albeit with limited patient numbers is the first evaluating patient satisfaction and cost benefit of domiciliary antibiotics in patients with NOE. All patients preferred to be treated at home with no safety issues raised. There is a potential saving of £2347 per patient with domiciliary rather than in-patient treatment as well as ‘freeing-up’ beds for other patients requiring admission.

**Table 1.** GBI scores for domiciliary versus hospital antibiotic treatment

	Total	General health	Social	Physical health
Range	8.3 to 22.2	16.6 to 29.1	−33.3 to 0	−50 to 33.3
Median	13.8	25	0	0
